# *SPOUT1* variants associated with autosomal-recessive developmental and epileptic encephalopathy

**DOI:** 10.1186/s42494-024-00185-0

**Published:** 2024-12-15

**Authors:** Wenwei Liu, Kai Gao, Xilong Du, Sijia Wen, Huifang Yan, Jingmin Wang, Yong Wang, Conglei Song, Li Lin, Taoyun Ji, Weiyue Gu, Yuwu Jiang

**Affiliations:** 1 Children’s Medical Center, Peking University First Hospital, Beijing, 100176 China; 2 Beijing Key Laboratory of Molecular Diagnosis and Study on Pediatric Genetic Diseases, Beijing, 100009 China; 3 Children Epilepsy Center, Peking University First Hospital, Beijing, 100176 China; 4 Key Laboratory for Neuroscience, Ministry of Education/National Health and Family Planning Commission, Peking University, Beijing, 100009 China; 5 Center of Epilepsy, Beijing Institute for Brain Disorders, Beijing, 100176 China; 6 Beijing Chigene Translational Medical Research Center Co. Ltd, Beijing, 101121 China; 7 Department of Pediatrics, Fujian Medical University Union Hospital, Fujian, 350001 China; 8 Department of Neurology, Anhui Children’s Hospital, Anhui, 230051 China

## Abstract

**Background:**

Developmental and epileptic encephalopathy (DEE) is a group of neurodevelopmental disorders characterized by early-onset seizures predominantly attributed to genetic causes. Nevertheless, numerous patients remain without identification of a genetic cause.

**Methods:**

We present four unrelated Chinese patients with *SPOUT1* compound heterozygous variants, all of whom were diagnosed with DEE. We also investigated functions of *SPOUT1* using the *spout1* knockout zebrafish model.

**Results:**

The four unrelated DEE patients with *SPOUT1* compound heterozygous variants were all males. Their onset age of seizure ranged from 3 months to 6 months (median age 5 months). All patients had epileptic spasms, and were diagnosed with infantile epileptic spasms syndrome (IESS). Three patients had microcephaly during infancy. Brain MRI in three patients showed white matter hypomyelination and bilaterally widened frontotemporal subarachnoid space. At the last follow-up, two patients exhibited drug-resistant epilepsy, one achieved seizure freedom following vigabatrin treatment, and one died at the age of 4 years and 5 months from probable sudden unexpected death in epilepsy. Seven different *SPOUT1* variants were identified in the four patients, including six missense variants and one deletion variant. AlphaFold2 prediction indicated that all variants alternated the number or the length of bonds between animo acids in protein SPOUT1. Neurophysiological results from *spout1* knockout zebrafish revealed the presence of epileptiform signals in 9 out of 63 *spout1* knockout zebrafishes (*P* = 0.009). Transcriptome sequencing revealed 21 differentially expressed genes between *spout1* knockout and control groups, including 13 up-regulated and 8 down-regulated genes. Two axonal transport-related genes, *kif3a* and *ap3d1*, were most prominently involved in enriched Gene Ontology (GO) terms.

**Conclusions:**

This study identified *SPOUT1* as a novel candidate gene of DEE, which follows the autosomal-recessive inheritance pattern. IESS is the most common epilepsy syndrome. Downregulation of axonal transport-related genes, *KIF3A* and *AP3D1*, may play a crucial role in the pathogenesis of DEE.

**Supplementary Information:**

The online version contains supplementary material available at 10.1186/s42494-024-00185-0.

## Background

Developmental and epileptic encephalopathy (DEE) is a group of heterogeneous disorders characterized by early-onset epilepsy, abnormal electroencephalography and developmental delay or regression, predominantly due to genetic factors [1]. DEE causes a high incidence of disability and mortality [2]. With the development of next-generation sequencing technology, 116 DEE-related genes have been discovered and are listed in the Online Mendelian Inheritance in Man database (OMIM). Nevertheless, a significant number of cases remain without definitive genetic explanations.

*SPOUT1*, also known as *CENP32* (Human), *C9orf114* (Human) or *D2Wsu81e* (Mouse), is located at chromosome 9q34.11, which was first reported by Ohta et al. in 2010 [3]. It encodes protein SPOUT domain-containing methyltransferase 1 (SPOUT1), which consists of 376 amino acids. *SPOUT1* is highly expressed in the embryonic stage and during childhood, and is widely present in multiple tissues and organs in humans. The function of *SPOUT1* is not clear yet. In 2015, Ohta et al. reported that it plays an important role in centrosome integration into a fully functional spindle, and could affect microtubule regrowth and prolong cell cycle [4]. In 2017, Treiber et al. reported that SPOUT1 may act as a methyltransferase to regulate posttranscriptional modification of RNA [5].

However, the associations of *SPOUT1* variants with diseases remain largely unknown. In 2014, Fromer et al. identified a heterozygous missense variant p.(Asp37Ala) of *SPOUT1* in 1 out of 623 schizophrenia patients [6]. In 2017, Reuter et al. reported two cousins from a consanguineous Israeli family who both carried the homozygous *SPOUT1* variant p.(Thr353Met) and were diagnosed with intellectual disability, seizures, microcephaly, short stature, limb hypertonia and bruxism [7]. 

In this study, we report four unrelated Chinese patients with *SPOUT1* compound heterozygous variants who were all diagnosed as DEE, and investigated the pathogenicity of *SPOUT1* in zebrafish. These results suggest *SPOUT1* as a new candidate pathogenic gene for DEE.

## Materials and methods

### Patients and clinical assessment

Children were enrolled at the Children’S Medical Center of Peking University First Hospital, Fujian Medical University Union Hospital, and Anhui Children’s Hospital from January 2016 to November 2023. Demographics, clinical manifestations, family history, genetic data, electroencephalography (EEG), neuroimaging, and therapeutic regimes of patients were collected from the clinic. Patients were followed-up at the pediatric neurology clinic or by telephone (Table 1).

**Table 1:** *SPOUT1* compound heterozygous variants in the four patients in our study

Case No.	Sex/Age	Gene variant	Protein variant	mRNA variant	Origin	MAF	SIFT	PolyPhen2	Provean	Mutation Taster	M-CAP	CADD	ACMG
1	Male 6y5m	c.598 C > T c.744_746delATC	p.Arg200Trp p.248_249delSer	NM_016390	M P	0.0001877 0.0000056	D(0.019) /	PD(0.993) /	D(-4.66) /	D(0.999996) /	D(0.030522) /	D(26.2) D(17.5)	VUS: PM2 + PP3 VUS: PM2
2	Male 11y9m	c.1046G > A c.662T > C	p.Arg349His p.Leu221Pro	NM_016390	M P	0.0000181 0.0000032	D(0.001) D(0.001)	PD(1.0) PD(1.0)	D(-4.45) D(-6.12)	D(0.999994) D(1)	D(0.065527) D(0.095759)	D(28.8) D(26.6)	VUS: PM2 + PP3 VUS: PM2 + PP3
3	Male 7y	c.1058 C > T c.1055G > A	p.Thr353Met p.Arg352His	NM_016390	M P	0.0000050 0.0000149	D(0.0) D(0.0)	PD(1.0) PD(0.935)	D(-5.46) D(-4.55)	D(0.99999) D(0.999935)	D(0.040058) D(0.097281)	D(25.8) D(24.3)	VUS: PM2 + PP3 VUS: PM2 + PP3
4	Male 4y5m	c.598 C > T c.625T > A	p.Arg200Trp p.Cys209Ser	NM_016390	M P	0.0001877 0	D(0.019) D(0.001)	PD(0.993) PD(0.996)	D(-4.66) D(-4.63)	D(0.999996) D(0.832828 )	D(0.030522) D(0.085996)	D(26.2) D(27.9)	VUS: PM2 + PP3 VUS: PM2 + PP3

*Abbreviation*: *M* Maternal, *P* Paternal, *D* Damaging, *PD* Probably damaging, *SIFT* Sorting Intolerant From Tolerant, *PolyPhen2* Polymorphism Phenotyping v2, *Provean* Protein Variation Effect Analyzer; *M-CAP* Mendelian Clinically Applicable Pathogenicity, *CADD* Combined Annotation Dependent Depletion, *ACMG* American College of Medical Genetics and Genomics, *VUS* Variant of unknown significance

This study was approved by the Institutional Review Board of the Ethics Committee of Peking University First Hospital (2005-004). Informed consent was obtained from parents of the children.

### Genetic analysis

Variant screening of *SPOUT1* (NM_016390, GRCh37/hg19) was performed using whole-exome sequencing or whole-genome sequencing from peripheral blood of probands and their parents. Pathogenicity of variants was interpreted according to the American College of Medical Genetics (ACMG) guidelines [8]. All missense variants were evaluated with the MutationTaster server (http://www.mutationtaster.org/), Polymorphism Phenotyping v2 (http://genetics.bwh.harvard.edu/pph2/), Protein Variation Effect Analyzer (https://www.jcvi.org/research/provean), Sorting Intolerant From Tolerant (http://sift.jcvi.org/), Mendelian Clinically Applicable Pathogenicity (http://bejerano.stanford.edu/mcap/), and Combined Annotation Dependent Depletion v1.7 (https://cadd.gs.washington.edu/download). Potential pathogenic variants were validated in Chigene (Beijing, China). Minor allele frequencies (MAFs) for all these variant sites were obtained from the Genome Aggregation Database, GnomAD (https://gnomad.broadinstitute.org).

### Homology modeling of the human SPOUT1

The human SPOUT1 chain used AlphaFold model AF-Q5T280-F1 as the template. And InterPro was used to predict the domain of protein SPOUT1. The protein structures of different variants of SPOUT1 protein were predicted by AlphaFold2 (https://github.com/deepmind/alphafold). The structures have been created by using the PyMOL Molecular Graphics System, Version 2.4.0a0.

### CRISPR-mediated knockout of *spout1 *in Zebrafish

The *SPOUT1* ortholog of zebrafish is *spout1* (ENSDARG00000019707). To generate *spout1* loss-of-function zebrafish, four single-guide RNAs (sgRNAs) were predicted by CHOPCHOP (http://chopchop.cbu.uib.no/) and evaluated by CRISPRater (https://cctop.cos.uni-heidelberg.de/). The sequences of the sgRNAs are as follows: ACAGTGAGCGTGGCTCTGCCTGG (sgRNA1), ACGCTCAGTCTCCAGAGCTGCGG (sgRNA2), TCCAGAGCTGCGGACGTATCTGG (sgRNA3), and GAGCTGCGGACGTATCTGGCTGG (sgRNA4). Zebrafish fertilized eggs were collected and injected with ~ 1 nl of CRISPR complexes composed of each sgRNA (90 ng/µL) and Cas9 protein (250 ng/µl). After 24 h, embryos were pooled and Sanger sequenced to verify the mutagenesis efficacy using the TIDE online tool (https://tide.nki.nl/). Two sgRNA sequences with the highest cleavage efficiency, sgRNA2 and sgRNA3, were selected. Each of them was mixed with Cas9 protein, and injected into zebrafish fertilized eggs for CRISPAR knockout of *spout1* in zebrafish.

### Zebrafish maintenance and breeding

Adult zebrafish were housed in circulating water at 28.5 °C under a 14/10-hour light/dark cycle and were fed twice daily. Zebrafish embryos were obtained through standard mating methods. Larvae were raised in a 28.5 °C incubator with E3 media composed of 0.03% sea salt and 0.00014% methylene blue in reverse osmosis-distilled water. All procedures were conducted following the Guideline for the Care and Use of Animals (2011).

### Electrophysiology of zebrafish

Cas9-control and *spout1*-knockout larvae aged 5–6 days post-fertilization (dpf) were used for electrophysiological recording. They were immobilized in 300 µM pancuronium (Sigma-Aldrich, Missouri, USA) and then embedded in 2% low-melting-point agarose in a recording chamber filled with embryo media. Local field potential (LFP) recording was made from the optic tectum using a glass microelectrode (1-µm diameter, 2–7 MΩ). Electrodes were filled with 2 M NaCl, and electrical activity was recorded using an extracellular amplifier. Data were low-pass filtered at 5 kHz, high-pass filtered at 1 Hz, and digitized at 10 kHz. Recordings were analyzed using the open-source software DClamp.

### RNA-Seq and transcriptome analysis

Briefly, RNA was extracted from 5 dpf zebrafish of each group (3 replicates per group). Then, mRNA was extracted by TIANSeq mRNA Capture Kit (TIANGEN, Beijing, China). The transcriptome sequencing libraries was constructed by the TIANSeq Fast RNA Library Kit (Illumina, CA, USA). Clustering of the index-coded samples was performed using the cBot Cluster Generation System with TruSeq PE Cluster Kit v3-cBot-HS (Illumina, CA, USA). The libraries were sequenced using an Illumina Xplus platform and 150-bp paired-end reads were generated.

Clean data were obtained after removing reads containing adapter and low-quality reads with Trimmomatic. Then, the clean data were aligned to the zebrafish reference genome GRCz11 using Hisat2 v2.0.5. Differential expression between the two groups was analyzed using the DESeq2 R package (1.16.1). Differently expressed genes (DEGs) were defined as those with an adjusted *P*-value less than 0.05 and an absolute value of log_2_ fold-change (logFC) greater than 1.

Gene Ontology (GO) enrichment analysis of DEGs was performed with the Database for Annotation, Visualization and Integrated Discovery (DAVID, https://david.ncifcrf.gov/). GO terms with *P*-value < 0.05 were considered as significantly enriched by DEGs. The volcano map was drawn by R language with ggplot2. The GOCircle and GOChord graphs were plotted by the GOplot package (1.0.2) in R.

### Statistics

Statistical analyses were performed using Prism 8 (GraphPad Prism Software, USA). Chi-square test was used for comparison of seizure frequency in zebrafish between groups. *P* < 0.05 was considered as statistically significant.

## Results

### Individuals with *SPOUT1* compound heterozygous variants display DEE

#### Genetic testing results

In our study, seven different *SPOUT1* variants were identified in four patients, all being compound heterozygous *SPOUT1* variants. One variant p.(Arg200Trp) was identified in two unrelated patients. Six variants were missense variants, including p.(Arg200Trp), p.(Cys209Ser), p.(Leu221Pro), p.(Arg349His), p.(Arg352His), and p.(Thr353Met), and the other one was deletion variant, p.(248_249delSer) (Table 1). The MAFs of all seven variants were below 0.001. The six missense variants were predicted to be damaging or probably damaging based on analyses with multiple bioinformatics tools. All seven variants were classified as variants of uncertain significance according to ACMG guidelines (Table 1).

**Fig. 1:**
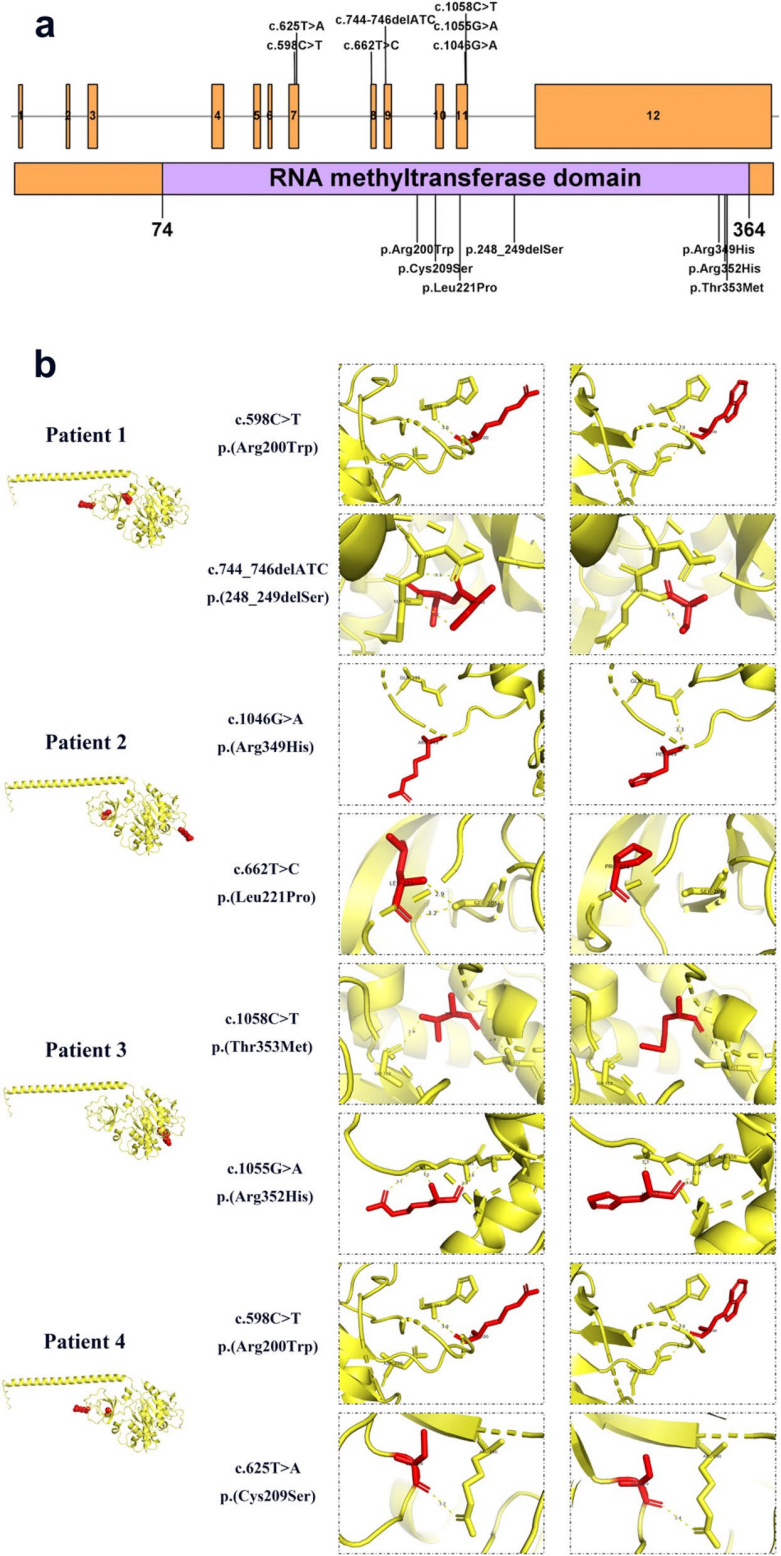
**a** *SPOUT1* gene, protein regions and variant sites. **b** Protein structures of different *SPOUT1* variants as predicted by AlphaFold

All of the *SPOUT1* variants were located within the RNA methyltransferase domain (Fig. 1a). All seven variants were predicted by AlphaFold2 to lead to alterations in the number or the length of bonds between amino acids (Fig. 1b).

#### Clinical features

Patients were all males. The four patients with *SPOUT1* compound heterozygous variants were from four different families. Three of them had normal family history. Patient 3 had an elder sister who was diagnosed with IESS (Fig. S12-S15). However, detailed clinical data for this patient was not obtained, and follow-up was lost. The age of seizure onset ranged from 3 to 6 months (median age, 5 months). Epileptic spasms were observed in all four patients, with only Patient 1 exhibiting focal seizures during the observation period. All four patients were diagnosed with global developmental delay, and failed to achieve age-appropriate gross motor milestones. Three patients presented with microcephaly during infancy (Table 2).

**Table 2:** Phenotypes of the four patients with *SPOUT1* compound heterozygous variants

Case No.	Sex/Age	Gene variant	Age onset	Family history	Clinical phenotype	DD	Microcephaly	VEEG	Brain MRI	Treatment and Prognosis
1	M/6 years and 5 months	c.598 C > T c.744_746delATC	6 months	Normal	IESS, hypertonia, nystagmus, eye adduction, PEM	GDD	OFC <-3SD	Background: / Interictal: Multifocal discharge, Hypsarrhythmia? Ictal: Epileptic spasms	5 months, 1 year and 3 months, 2 years and 11 months: ACC; 2 years: Bilaterally widened frontotemporal subarachnoid space, bilateral frontal atrophy; White matter hypomyelination	Refractory: TPM, VAP (ACTH, VitB6, VGB, LEV, KD)
2	M/11 years and 9 months	c.1046G > A c.662T > C	5 months	Normal	IESS, hypertonia	GDD	N	Background: / Interictal: Atypical hypsarrhythmia; Ictal: Epileptic spasms	6 months: N	Seizure free (ACTH, VitB6, VGB)
3	M/7 years	c.1058 C > T c.1055G > A	5 months	Elder sister diagnosed as IESS	IESS	GDD	OFC <-3SD	Background: / Interictal: Atypical hypsarrhythmia, Multifocal discharge; Ictal: /	5 months: Bilaterally widened frontotemporal subarachnoid space; White matter hypomyelination	Refractory: VGB, VPA, CZP (LEV, ACTH, VitB6)
4	M/4 years and 5 months (D)	c.598 C > T c.625T > A	3 months	Normal	IESS, SUDEP	GDD	OFC <-3SD	Background: Slow; Interictal: Atypical hypsarrhythmia, Multifocal discharge; Ictal: Epileptic spasms	4 months: Bilaterally widened frontotemporal subarachnoid space; White matter hypomyelination	SUDEP (VPA, LEV, TPM, ACTH)

*Abbreviation*: *M* Male, *D* Death, *N* Normal, *IESS* Infantile epileptic spasms syndrome, *PEM* Protein–energy malnutrition, *SUDEP* Sudden unexpected death in epilepsy, *GDD* Global developmental delay, *OFC* Occipital frontal circumference, *ACC* Agenesis of corpus callosum, *VPA* Valproate, *LEV* Levetiracetam, *TPM* Topiramate, *CZP* Clonazepam, *VGB* Vigabatrin, *ACTH* Adrenocorticotropic hormone. Drugs in parentheses were prior drugs

#### Video electroencephalography (VEEG)

**Fig. 2:**
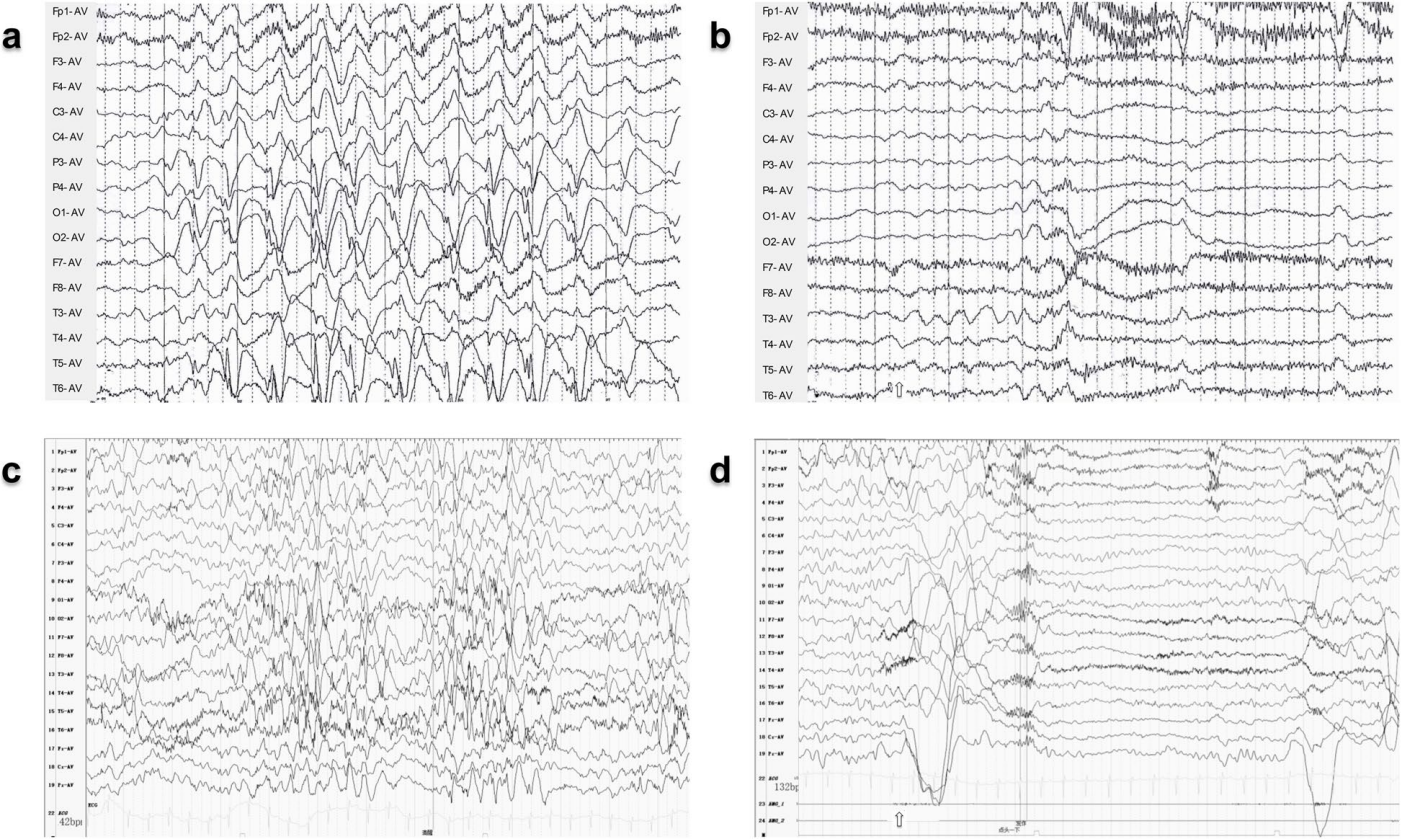
Electroencephalography (EEG) of patients with *SPOUT1 *variant. Interictal EEG and ictal scalps of Patient 2 (**a**, **b**) and Patient 4 (**c**, **d**) demonstrating atypical hypsarrhythmia (**a**, **c**) and monitored epileptic spasms (**b**, **d**)

All patients exhibited abnormalities on VEEG. Patient 4 demonstrated a background characterized by diffuse slow activity with low voltage. Interictal EEG recordings in three patients revealed atypical hypsarrhythmia, while one patient exhibited hypsarrhythmia (record lost). Multifocal discharges were observed in two patients (Patients 1 and 4). During EEG monitoring, seizures were recorded in three patients (Patients 1, 2 and 4), all of whom experienced epileptic spasms (Table 2; Fig. 2).

#### Neuroimaging

**Fig. 3:**
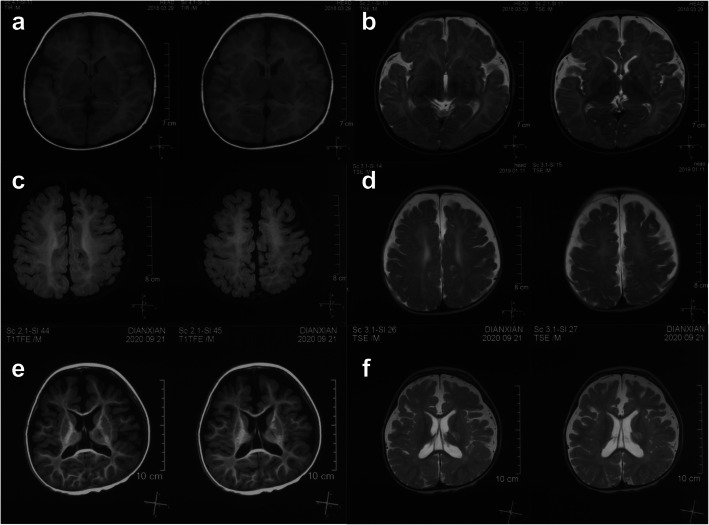
Brain MRI of Patient 1 at age of 5 months (**a**, **b**), 1 year and 3 months (**c**, **d**) and 2 years and 11 months (**e**, **f**). Agenesis of corpus callosum, bilateral frontal atrophy, and white matter hypomyelination were shown

Brain MRI scans revealed white matter hypomyelination and bilaterally widened frontotemporal subarachnoid spaces in three patients (Patients 1, 3 and 4). One patient demonstrated bilateral frontal atrophy over time. Patient 1 exhibited agenesis of the corpus callosum (Table 2; Fig. 3). Patient 2 had normal MRI findings.

#### Epileptic syndrome

The four patients were diagnosed with infantile epileptic spasms syndrome (IESS) (Table 2). All had an age of seizure onset before 6 months and exhibited delays in age-appropriate gross motor milestones prior to the onset of seizures. Consequently, they all could be diagnosed with DEE.

#### Treatment and prognosis

The age of the patients at the final follow-up ranged from 4 years and 5 months to 11 years and 9 months. At the last follow-up, two patients exhibited drug-resistant epilepsy (Patients 1 and 3), and one patient achieved seizure freedom after vigabatrin treatment, and has been off from anti-seizure medication for 8 years (Patient 2). Patient 4 died at age of 4 years and 5 months, due to probable sudden unexpected death in epilepsy (SUDEP).

### Knockout of *spout1* increases epileptic discharges in zebrafish

**Fig. 4:**
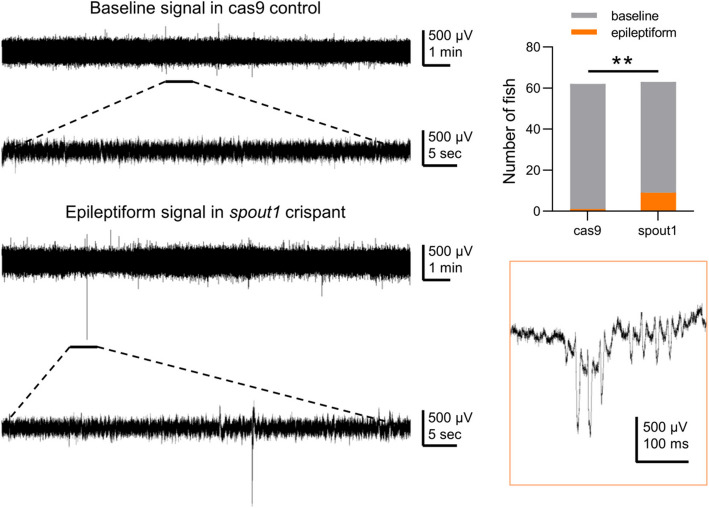
Representative electrophysiological recordings and statistical analysis of zebrafish between the cas9-control group and *spout1*-knockout group. **P < 0.01

To assess the role of *SPOUT1* in neurophysiology, we knocked out *spout1* in zebrafish and recorded field potentials in the optic tectum of zebrafish at 5–6 days dpf. We detected epileptiform-like signals in only 1 of 62 Cas9-control zebrafish. In contrast, in the *spout1*-knockout group, 9 out of 63 zebrafish exhibited epileptiform signals, which was significantly different from the Cas9 control group (Chi-square test, *P* = 0.009; Fig. 4). In summary, our findings suggest that knockout of *spout1* in zebrafish increased abnormal EEG epileptic discharges, supporting the association between *spout1* gene variants and epilepsy (Fig. S1-S11).

### Knockout of *spout1* may lead to the downregulation of axonal transport-related genes *KIF3A* and *AP3D1*

**Fig. 5:**
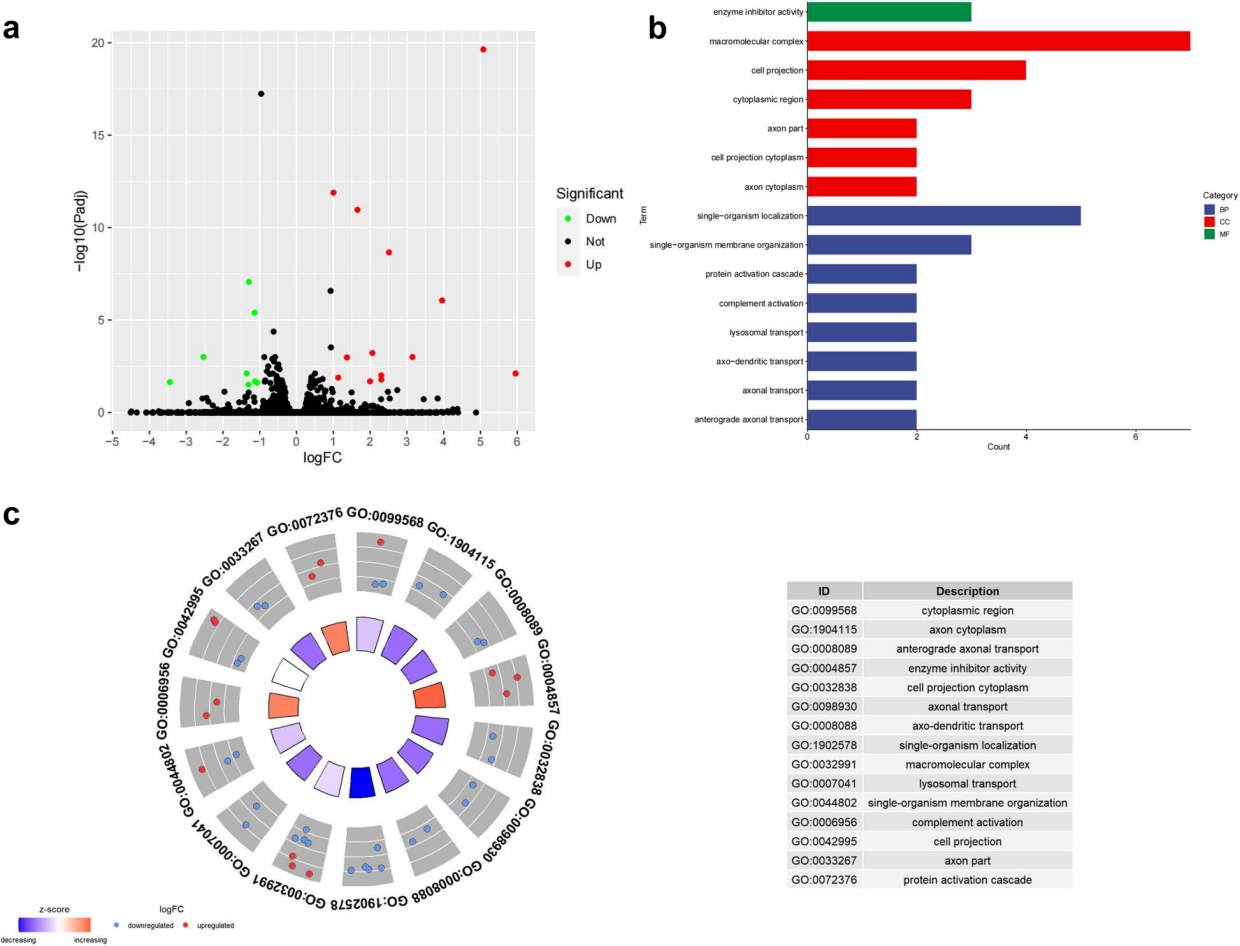
RNAseq analysis of *spout1*-knockout zebrafish.** a** Volcano map of gene expression changes after *spout1* knockout. Red dots represent up-regulated genes. Green dots represent downregulated genes. **b** Significantly enriched GO terms, which were grouped into three categories: molecular function (MF), cellular component (CC) and biological process (BP). **c** GOCircle plot based on the GOplot analysis of enriched GO term. The inner ring is a barplot where the height of the bar indicates the significance of the GO term (-log10 |*P*-value|), and color corresponds to the *z*-score, which indicates increase (red) or decrease (blue). The outer ring displays scatterplots of the expression levels (logFC) of the genes in each term

To identify the DEGs in *spout1*-knockout zebrafish, transcriptome sequencing was performed at 5 dpf. RNA-seq identification of DEGs was performed with Hisat2 and DESeq2. A total of 21 genes were differentially expressed, including 13 up-regulated genes and 8 down-regulated genes (Table 3; Fig. 5a). Volcano map showed that potassium ion channel gene *kcnj1a.6*, gamma-aminobutyric acid (GABA) A receptor gene *gabrp*, and synapse-related gene *sybl1*, were down-regulated.

**Table 3:** Significant differentially expressed genes in *spout1-*knockout zebrafish

Gene ID	Gene name	log_2_FC	*P* value	*P* adj	Trend	Description
ENSDARG00000074983	*jac9*	5.077020026	1.34E-24	2.31E-20	UP	Jacalin 9
ENSDARG00000100513	*rps27l*	1.002846741	1.48E-16	1.28E-12	UP	Ribosomal protein S27 like
ENSDARG00000087359	*c3a.2*	1.657808476	1.59E-15	1.10E-11	UP	Complement component c3a, duplicate 2
ENSDARG00000096603	*bmb*	2.511565265	3.79E-13	2.19E-09	UP	Brambleberry
ENSDARG00000105183	*CT009487.2*	-1.294186345	1.76E-11	8.68E-08	DOWN	
ENSDARG00000076958	*jac8*	3.957651611	2.28E-10	8.76E-07	UP	Jacalin 8
ENSDARG00000071424	*ap3d1*	-1.140269129	1.15E-09	3.97E-06	DOWN	Adaptor related protein complex 3 subunit delta 1
ENSDARG00000020901	*gabrp*	2.061180696	2.29E-07	0.000608241	UP	Gamma-aminobutyric acid (GABA) A receptor, pi
ENSDARG00000030775	*sybl1*	-2.532133395	4.32E-07	0.000996733	DOWN	Synaptobrevin-like 1
ENSDARG00000097929	*BX005392.3*	3.149784541	5.16E-07	0.001005299	UP	si: dkey-90a24.1
ENSDARG00000103650	*si: ch73-329n5.1*	1.371213395	5.78E-07	0.001052966	UP	si: ch73-329n5.1
ENSDARG00000040738	*zgc:153846*	-1.355794033	7.46E-06	0.007753712	DOWN	zgc:153846
ENSDARG00000094508	*CR925709.2*	5.954715441	7.67E-06	0.007753712	UP	
ENSDARG00000033126	*prkrip1*	2.303220779	1.06E-05	0.009904536	UP	PRKR interacting protein 1
ENSDARG00000038424	*c4b*	1.133160953	1.51E-05	0.013090889	UP	Complement 4B (Chido blood group)
ENSDARG00000070735	*rnd2*	2.305009446	2.26E-05	0.016670917	UP	Rho family GTPase 2
ENSDARG00000016710	*rchy1*	1.998077888	3.41E-05	0.020641378	UP	RINg finger and CHY zinc finger domain containing 1
ENSDARG00000088484	*kcnj1a.6*	-1.133963075	3.46E-05	0.020641378	DOWN	Potassium inwardly rectifying channel subfamily J member 1a, tandem duplicate 6
ENSDARG00000019763	*acp5a*	-3.441415222	4.05E-05	0.02296736	DOWN	Acid phosphatase 5a, tartrate resistant
ENSDARG00000019707	*spout1*	-1.075043145	4.46E-05	0.024473222	DOWN	SPOUT domain containing methyltransferase 1
ENSDARG00000087538	*kif3a*	-1.305141748	6.52E-05	0.031338507	DOWN	kinesin family member 3 A

To further evaluate the biological functions of these DEGs, GO enrichment analysis was performed. Results showed that the DEGs belonged to 15 GO terms, grouped into three categories: molecular function (MF), cellular component (CC) and biological process (BP) (Fig. 5b). The BP category included GO:0008089 anterograde axonal transport, GO:0098930 axonal transport, GO:0008088 axo-dendritic transport, GO:1902578 single-organism localization, GO:0007041 lysosomal transport, GO:0044802 single-organism membrane organization, GO:0006956 complement activation, and GO:0072376 protein activation cascade. The MF category was GO:0004857 enzyme inhibitor activity. In the cellular component, GO:0099568 cytoplasmic region, GO:1904115 axon cytoplasm, GO:0032838 cell projection cytoplasm, GO:0032991 macromolecular complex, GO:0042995 cell projection, and GO:0033267 axon part.

It should be mentioned that GO:1902578, GO:1904115, GO:0008089, GO:0032838, GO:0098930, GO:0008088, and GO:0033267 were negatively regulated as most of their DEGs were down-regulated. On the other hand, GO:0004857, GO:0072376 and GO:0006956 were positively regulated because most of their DEGs were up-regulated (Fig. 5c). Of the DEGs, there were two key genes, *kif3a* and *ap3d1*, which were most prominently involved in the enriched GO terms (Fig. 5c). This observation may indicate that genes *KIF3A* and *AP3D1* play an important role in the pathogenesis of *SPOUT1* variant related DEE.

## Discussion

The concept of DEE was proposed by International League Against Epilepsy (ILAE) in 2017 [1]. Although genetic inheritance is the most common etiology of DEE, a substantial number of patients diagnosed with DEE do not have identifiable pathogenic genes. Recent studies have reported that DEE caused by different genes may exhibit distinct characteristics [9].

IESS is a prevalent clinical epilepsy syndrome of DEE, which has been associated with over 50 genes. Notably, IESS associated with over 90% of these genes demonstrate an autosomal-dominant inheritance pattern. A limited number of genes, including *PARS2*, *BUA5*, *WWOX*, *SLC25A22*, *SZT2*, *UGDH*, and *ACTL6B*, have been reported to follow an autosomal-recessive inheritance pattern [10–12]. In our study, we identified four independent IESS patients carrying compound heterozygous variants of *SPOUT1*. Based on previous literature, we hypothesize that *SPOUT1*-related DEE (*SPOUT1*-DEE) adheres to an autosomal-recessive inheritance pattern [7].

Our four patients with *SPOUT1* compound heterozygous variants had similar clinical phenotypes. Their seizure-onset age were below 6 months. The most common seizure type was epileptic spasms, and the most common epilepsy syndrome was IESS. Most of the patients (3/4) had microcephaly. Hypertonia, nystagmus, and protein-energy malnutrition might be the manifestations of *SPOUT1*-related patients. The neuroimaging features of *SPOUT1*-DEE included white matter hypomyelination and bilaterally widened frontotemporal subarachnoid spaces in three patients, as well as agenesis of corpus callosum in one patient. One patient exhibited normal brain MRI, potentially due to the younger age. The death of one patient suggests that patients with *SPOUT1* variants might have a risk of SUDEP.

A missense variant p.(Arg200Trp) was identified in two independent patients (Patients 1 and 4), who had a seizure onset age of 6 months and 3 months, respectively. Both of them were diagnosed with IESS and microcephaly during infancy. Despite treatment with multiple anti-seizure medications, they continued to experience refractory epilepsy. Their brain MRIs consistently demonstrated white matter hypomyelination and bilateral frontal atrophy. Patient 4 even succumbed to SUDEP at age of 4 years.

In our four patients, we identified seven distinct *SPOUT1* variants, predominantly missense variants, with one being deletion variant. MAFs of all these variants were below 0.001. Bioinformatics analyses suggest that all identified variants had the potential to alter the structure and function of SPOUT1 protein. SPOUT1 is known to function as a methyltransferase involved in RNA posttranscriptional modification [5]. Notably, *SPOUT1* variants identified in our four patients were located within the RNA methyltransferase domain. Consequently, we hypothesize that the *SPOUT1* variants may impair the function of the protein by disrupting RNA methylation processes.

We further studied *SPOUT1* function in *spout1-*knockout zebrafish. Compared to mice that have close physiological and genetic similarity with humans, zebrafish models have advantages in terms of the time cost, high-throughput screening, genetic manipulation, and phenotype display for the research of epilepsy. Recently, a series of investigations have employed zebrafish as an excellent model of epilepsy, to study the mechanisms of epileptogenesis and strategies of epilepsy therapy [13–15]. To confirm the relationship between *SPOUT1* and epilepsy, we constructed a CRISPR-mediated *spout1* knockout zebrafish model. The *spout1*-knockout zebrafish exhibited significant changes in neural electrophysiology. Following the knockout of *spout1*, zebrafish exhibited atypical epileptiform discharges, a discharge pattern that was infrequently observed in wild-type zebrafish. This study demonstrated for the first time that the knockout of *spout1* could increase epileptic discharges in zebrafish, speculating that it might be involved in the pathogenesis of epilepsy.

Previous studies have shown that a deletion variant of *spout1* in chicken DT4 cells did induce a mild growth defect [4]. In our study, we found that knockout of *spout1* could increase epileptic discharges in zebrafish. Combined with clinical phenotype spectrum of *SPOUT1*-DEE patients, we predicted that *SPOUT1* variants may potentially result in loss-of-function.

To further investigate the function of *SPOUT1*, we performed transcriptome sequencing and demonstrated that knockout of *spout1* could potentially perturb the development of the nervous system and neural electrophysiology through multiple pathways. A principal effect observed was the alteration of intracellular single-organism localization (GO:1902578), particularly in axonal transport (GO:0098930). This alteration appeared to be mediated by the down-regulated genes *kif3a* and *ap3d1*. KIF3A is a microtubule-related anterograde motor in axons [16], and its loss of function leads to abnormal neuronal migration and differentiation, thereby resulting in brain development anomalies in humans [17]. *AP3D1* variants have been linked to epilepsy [18]. Knockout of *spout1* also induced upregulation of complement activation-related genes *c4b* and *c3a.2*. In humans, *C4B* has been implicated in Alzheimer’s disease [19], indicating that *C4B* might be involved in brain degeneration. Although some genes involved in signaling pathways such as the potassium ion channel gene *kcnj1a.6* and the gamma-aminobutyric acid A receptor gene *gabrp*, were down-regulated in *spout1-*knockout zebrafish, their homologues in humans are either not expressed in the brain or are pseudogenes, suggesting a low likelihood of their contribution to brain development disorders and epilepsy. Collectively, our data suggest that *spout1* knockout may lead to the downregulation of axonal transport-related genes *kif3a* and *ap3d1*, potentially disturbing axonogenesis and neurodevelopment, resulting in DEE. Future studies will be necessary to validate these findings.

### Limitation

This study had some limitations. First, the sample size was not large enough. Larger cohorts of *SPOUT1*-related DEE patients should be employed in future studies. Second, functional analyses for the seven identified *SPOUT1* variants remain incomplete. Third, animals differ significantly from humans, thus animal models cannot accurately represent all human characteristics. In future studies, alternative animal models, such as mice, should be used to investigate the function of *SPOUT1*. Last, there are inherent biological differences between zebrafish and humans. In the further, evidence from human subjects is needed to validate these findings.

## Conclusions

*SPOUT1*-DEE follows an autosomal recessive inheritance pattern. This condition typically manifests in early infancy, and is characterized by IESS. Most of the patients can be diagnosed with microcephaly. Neuroimaging features of *SPOUT1*-DEE often include white matter hypomyelination and bilaterally widened frontotemporal subarachnoid spaces, which may progress to cerebral atrophy over time. The presence of *SPOUT1* variants may also have implications for patient longevity. *Spout1* variants might lead to loss of function. *Spout1* knockout zebrafish exhibit increased epileptic discharges, accompanied by reduced expression of axonal transport-related genes *kif3a* and *ap3d1*. This downregulation potentially disrupts axonogenesis and neurodevelopment, ultimately exacerbating epileptic discharges.

## Supplementary Information

Supplementary Material 1.

## Data Availability

Not applicable.

## Abbreviations

ACMG: American College of Medical Genetics and Genomics
BP: Biological process
CC: Cellular component
DEE: Developmental and epileptic encephalopathy
DEGs: Differently expressed genes
GO: Gene Ontology
MAFs: Minor allele frequencies
MF: Molecular function
IESS: Infantile epileptic spasms syndrome
SUDEP: Sudden unexpected death in epilepsy
VEEG: Video electroencephalography

## References

[1] Scheffer IE, Berkovic S, Capovilla G, Connolly MB, French J, Guilhoto L, et al. ILAE classification of the epilepsies: position paper of the ILAE Commission for Classification and terminology. Epilepsia. 2017;58(4):512–21. 28276062 10.1111/epi.13709 PMC5386840

[2] Hamdan FF, Myers CT, Cossette P, Lemay P, Spiegelman D, Laporte AD, et al. High rate of recurrent De Novo mutations in Developmental and epileptic encephalopathies. Am J Hum Genet. 2017;101(5):664–85. 29100083 10.1016/j.ajhg.2017.09.008 PMC5673604

[3] Ohta S, Bukowski-Wills JC, Sanchez-Pulido L, Alves Fde L, Wood L, Chen ZA, et al. The protein composition of mitotic chromosomes determined using multiclassifier combinatorial proteomics. Cell. 2010;142(5):810–21. 20813266 10.1016/j.cell.2010.07.047 PMC2982257

[4] Ohta S, Wood L, Toramoto I, Yagyu K, Fukagawa T, Earnshaw WC. CENP-32 is required to maintain centrosomal dominance in bipolar spindle assembly. Mol Biol Cell. 2015;26(7):1225–37. 25657325 10.1091/mbc.E14-09-1366 PMC4454171

[5] Treiber T, Treiber N, Plessmann U, Harlander S, Daiß JL, Eichner N, et al. A compendium of RNA-Binding proteins that regulate MicroRNA Biogenesis. Mol Cell. 2017;66(2):270-e284213. 28431233 10.1016/j.molcel.2017.03.014

[6] Fromer M, Pocklington AJ, Kavanagh DH, Williams HJ, Dwyer S, Gormley P, et al. De novo mutations in schizophrenia implicate synaptic networks. Nature. 2014;506(7487):179–84. 24463507 10.1038/nature12929 PMC4237002

[7] Reuter MS, Tawamie H, Buchert R, Hosny Gebril O, Froukh T, Thiel C, et al. Diagnostic Yield and Novel candidate genes by Exome sequencing in 152 consanguineous families with Neurodevelopmental disorders. JAMA Psychiatry. 2017;74(3):293–9. 28097321 10.1001/jamapsychiatry.2016.3798

[8] Richards S, Aziz N, Bale S, Bick D, Das S, Gastier-Foster J, et al. Standards and guidelines for the interpretation of sequence variants: a joint consensus recommendation of the American College of Medical Genetics and Genomics and the Association for Molecular Pathology. Genet Med. 2015;17(5):405–24. 25741868 10.1038/gim.2015.30 PMC4544753

[9] Zuberi SM, Wirrell E, Yozawitz E, Wilmshurst JM, Specchio N, Riney K, et al. ILAE classification and definition of epilepsy syndromes with onset in neonates and infants: position statement by the ILAE Task Force on Nosology and definitions. Epilepsia. 2022;63(6):1349–97. 35503712 10.1111/epi.17239

[10] Nagarajan B, Gowda VK, Yoganathan S, Sharawat IK, Srivastava K, Vora N, et al. Landscape of genetic infantile epileptic spasms syndrome-A multicenter cohort of 124 children from India. Epilepsia Open. 2023;8(4):1383–404. 37583270 10.1002/epi4.12811 PMC10690684

[11] Chopra SS. Infantile spasms and West Syndrome - A Clinician’s perspective. Indian J Pediatr. 2020;87(12):1040–6. 32557136 10.1007/s12098-020-03279-y

[12] Pavone P, Polizzi A, Marino SD, Corsello G, Falsaperla R, Marino S, et al. West syndrome: a comprehensive review. Neurol Sci. 2020;41(12):3547–62. 32827285 10.1007/s10072-020-04600-5 PMC7655587

[13] Gawel K, Langlois M, Martins T, van der Ent W, Tiraboschi E, Jacmin M, et al. Seizing the moment: zebrafish epilepsy models. Neurosci Biobehav Rev. 2020;116:1–20. 32544542 10.1016/j.neubiorev.2020.06.010

[14] D’Amora M, Galgani A, Marchese M, Tantussi F, Faraguna U, De Angelis F, et al. Zebrafish as an innovative Tool for Epilepsy modeling: state of the art and potential future directions. Int J Mol Sci. 2023;24(9):7702. 37175408 10.3390/ijms24097702 PMC10177843

[15] Yaksi E, Jamali A, Diaz Verdugo C, Jurisch-Yaksi N. Past, present and future of zebrafish in epilepsy research. Febs j. 2021;288(24):7243–55. 33394550 10.1111/febs.15694

[16] Kondo S, Sato-Yoshitake R, Noda Y, Aizawa H, Nakata T, Matsuura Y, et al. KIF3A is a new microtubule-based anterograde motor in the nerve axon. J Cell Biol. 1994;125(5):1095–107. 7515068 10.1083/jcb.125.5.1095 PMC2120052

[17] Chen JL, Chang CH, Tsai JW. Gli2 rescues delays in Brain Development Induced by Kif3a Dysfunction. Cereb Cortex. 2019;29(2):751–64. 29342244 10.1093/cercor/bhx356

[18] Ammann S, Schulz A, Krägeloh-Mann I, Dieckmann NM, Niethammer K, Fuchs S, et al. Mutations in AP3D1 associated with immunodeficiency and seizures define a new type of Hermansky-Pudlak syndrome. Blood. 2016;127(8):997–1006. 26744459 10.1182/blood-2015-09-671636 PMC7611501

[19] Trouw LA, Nielsen HM, Minthon L, Londos E, Landberg G, Veerhuis R, et al. C4b-binding protein in Alzheimer’s disease: binding to Abeta1-42 and to dead cells. Mol Immunol. 2008;45(13):3649–60. 18556068 10.1016/j.molimm.2008.04.025

