# Correction: *SPOUT1* variants associated with autosomal-recessive developmental and epileptic encephalopathy

**DOI:** 10.1186/s42494-025-00240-4

**Published:** 2025-09-12

**Authors:** Wenwei Liu, Kai Gao, Xilong Du, Sijia Wen, Huifang Yan, Jingmin Wang, Yong Wang, Conglei Song, Li Lin, Taoyun Ji, Weiyue Gu, Yuwu Jiang

**Affiliations:** 1 Children’s Medical Center, Peking University First Hospital, Beijing, 100176 China; 2 Beijing Key Laboratory of Molecular Diagnosis and Study on Pediatric Genetic Diseases, Beijing, 100009 China; 3 Children Epilepsy Center, Peking University First Hospital, Beijing, 100176 China; 4 Key Laboratory for Neuroscience, Ministry of Education/National Health and Family Planning Commission, Peking University, Beijing, 100009 China; 5 Center of Epilepsy, Beijing Institute for Brain Disorders, Beijing, 100176 China; 6 Beijing Chigene Translational Medical Research Center Co. Ltd, Beijing, 101121 China; 7 Department of Pediatrics, Fujian Medical University Union Hospital, Fujian, 350001 China; 8 Department of Neurology, Anhui Children’s Hospital, Anhui, 230051 China

**Correction: Acta Epileptologica 6, 42 (2024)**

**https://doi.org/10.1186/s42494-024-00185-0**

Following publication of the original article [1], the authors reported an error in “Materials and methods” section, in subsection “CRISPR-mediated knockout of *spout1* in Zebrafish", the sequences of the sgRNAs ACGCTCAGTCTCCAGAGCTGCGG (sgRNA2), ACGCTCAGTCTCCAGAGCTGCGG (sgRNA3) should be corrected to ACGCTCAGTCTCCAGAGCTGCGG (sgRNA2), TCCAGAGCTGCGGACGTATCTGG (sgRNA3).

The original article [1] has been updated.
