# Supplementary material for: SPOUT1 variants associated with autosomal-recessive developmental and epileptic encephalopathy
Source: Acta Epileptol. 2024 Dec 15;6:42. doi: 10.1186/s42494-024-00185-0 (PMC11960386; doi:10.1186/s42494-024-00185-0)
Supplement: Supplementary file 1 — Supplementary Material 1. [file 42494_2024_185_MOESM1_ESM.docx]

**Supplemental data**


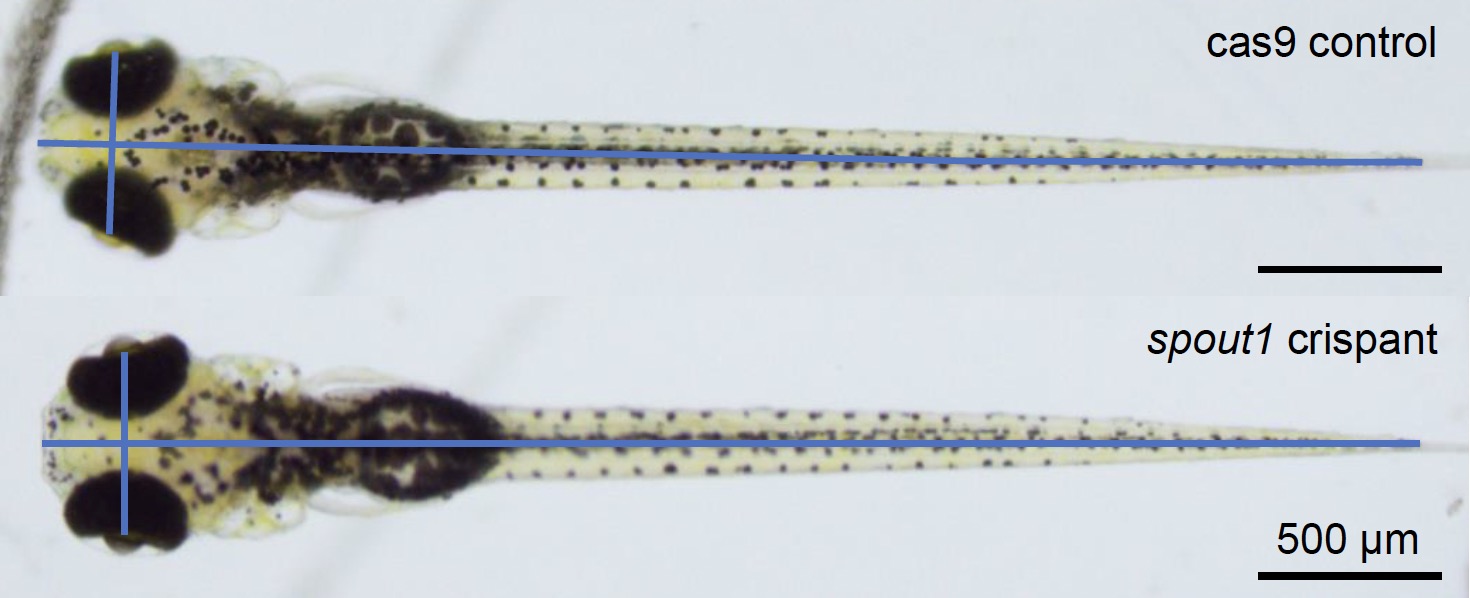


Fig. S1: Body length and eye distance between zebrafish in the cas9 control group and *spout1* knockout group.


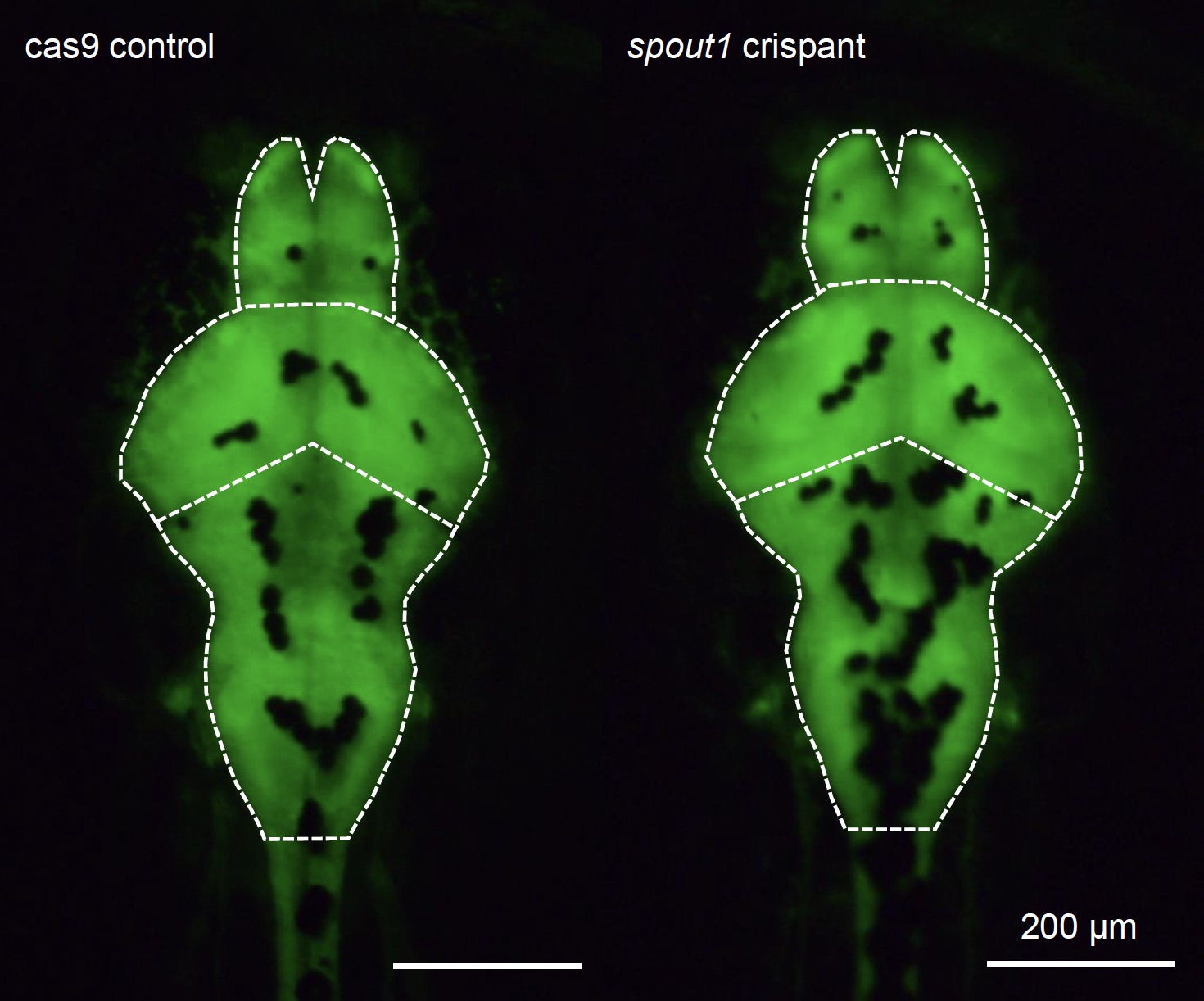


Fig. S2: Fluorescence photograph of central nerve system between zebrafish in the cas9 control group and *spout1* knockout group.


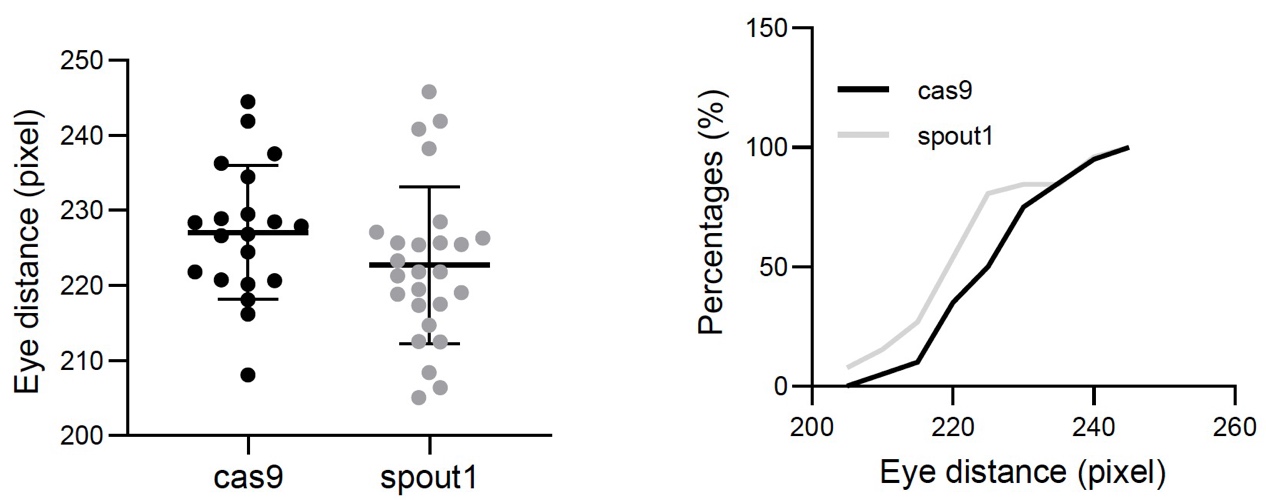


Fig. S3: Statistic analyses of eye distance between zebrafish in the cas9 control group and *spout1* knockout group.


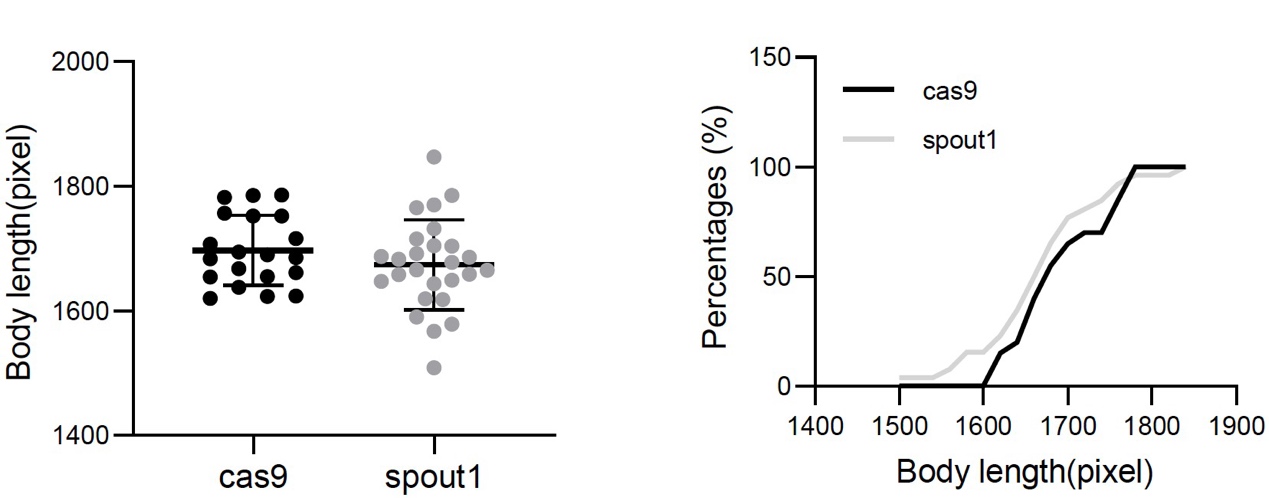


Fig. S4: Statistic analyses of body length between zebrafish in the cas9 control group and *spout1* knockout group.


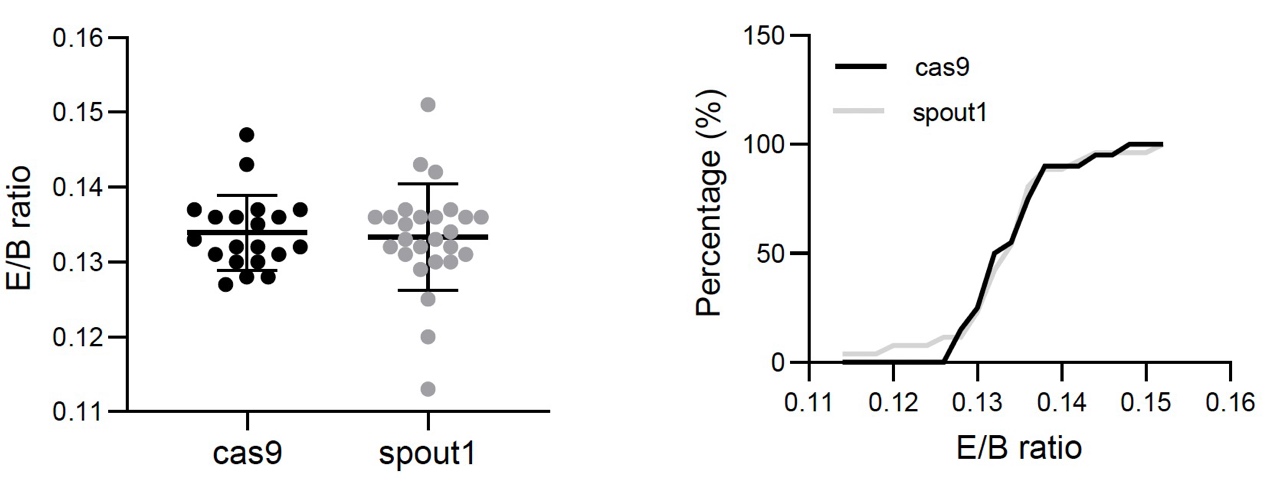


Fig. S5: Statistic analyses of E/B (eye distance/body length) ratio between zebrafish in the cas9 control group and *spout1* knockout group.


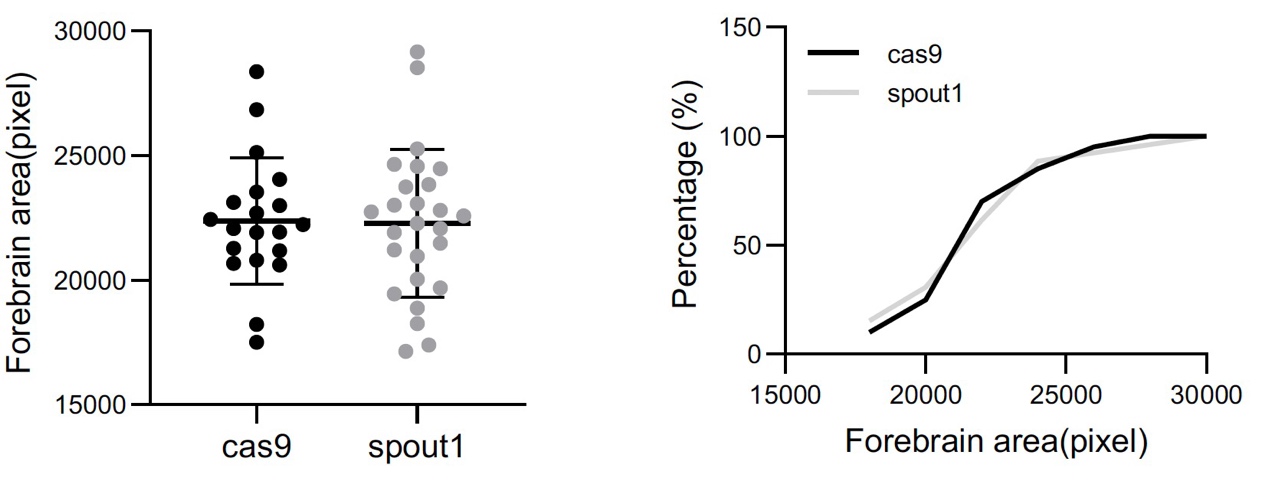


Fig. S6: Statistic analyses of forebrain area between zebrafish in the cas9 control group and *spout1* knockout group.


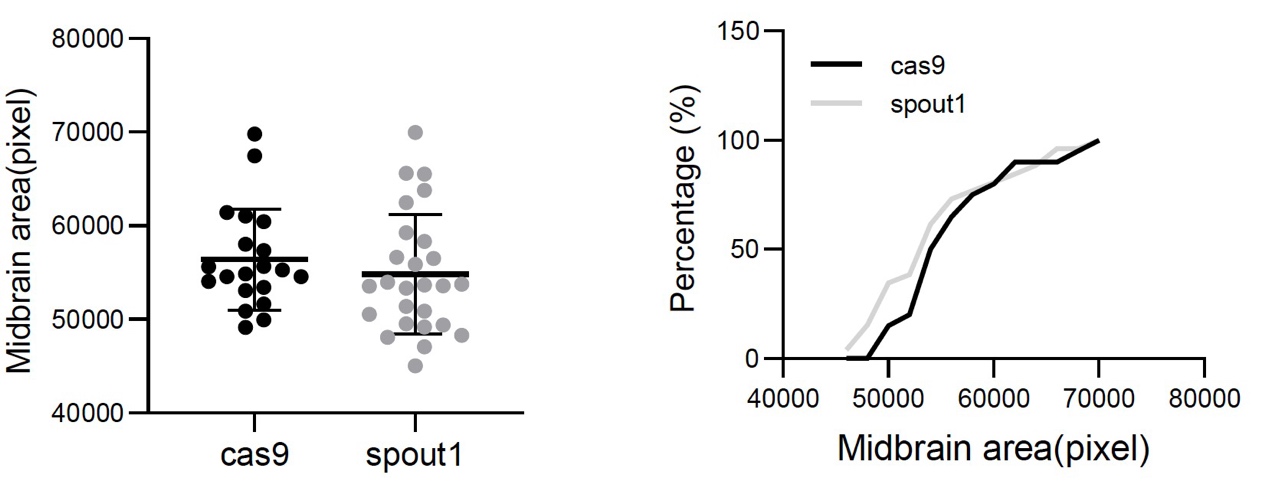


Fig. S7: Statistic analyses of midbrain area between zebrafish in the cas9 control group and *spout1* knockout group.


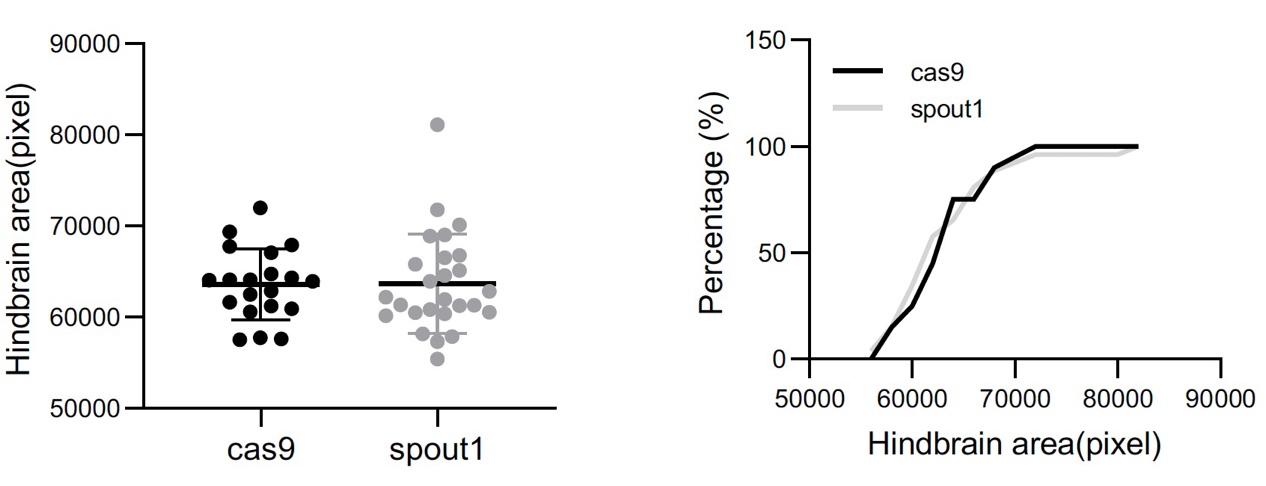


Fig. S8: Statistic analyses of hindbrain area between zebrafish in the cas9 control group and *spout1* knockout group.


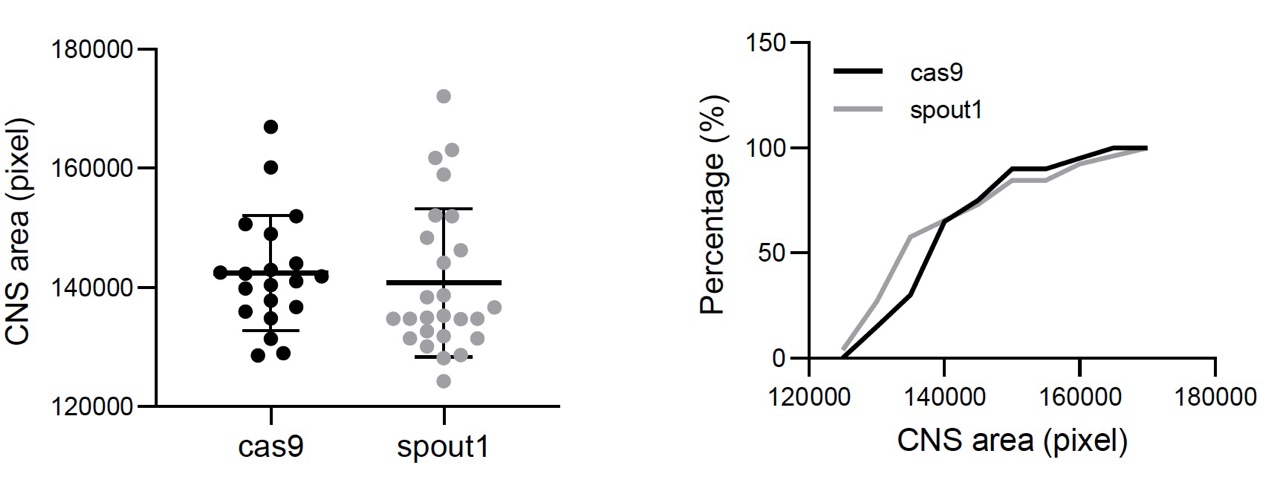


Fig. S9: Statistic analyses of central nerve system (CNS) area between zebrafish in the cas9 control group and *spout1* knockout group.


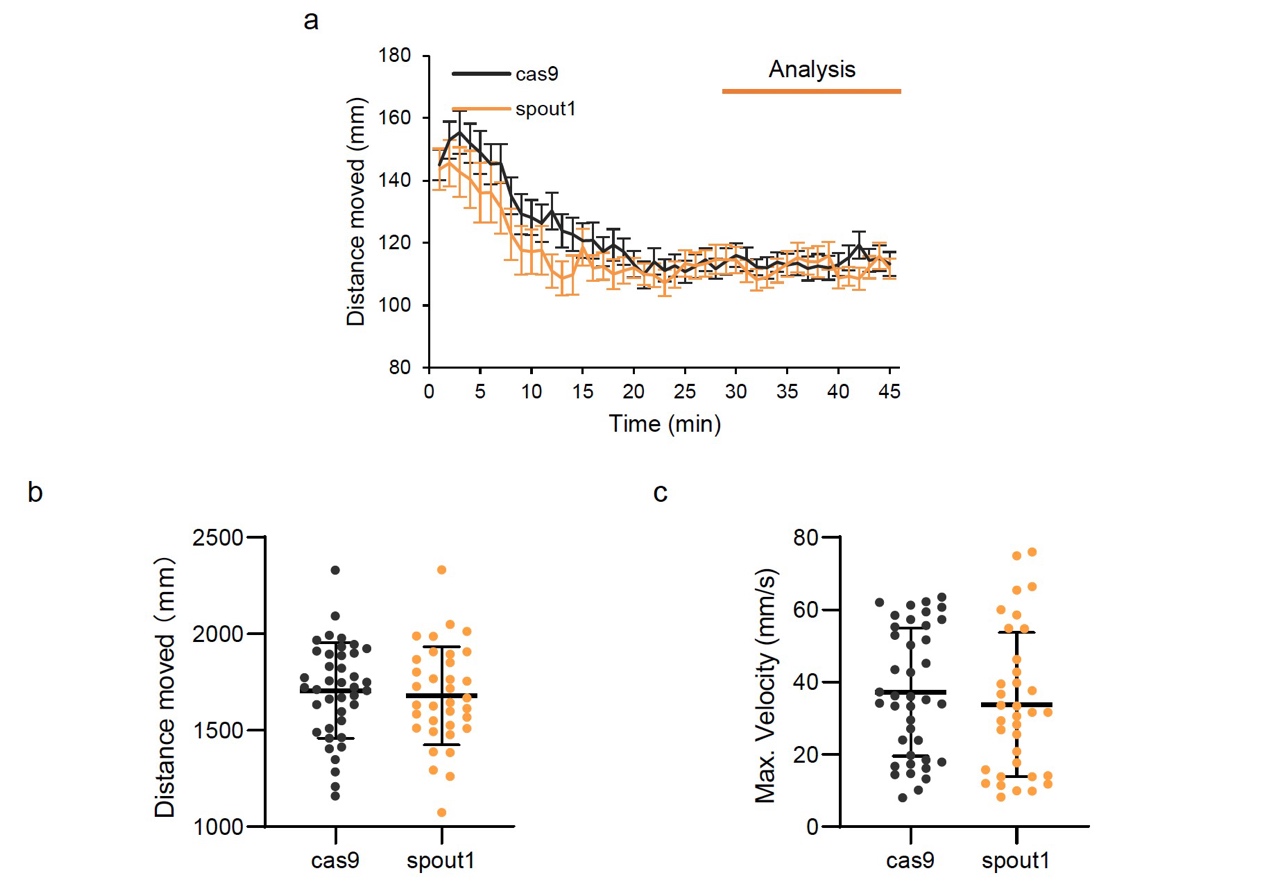


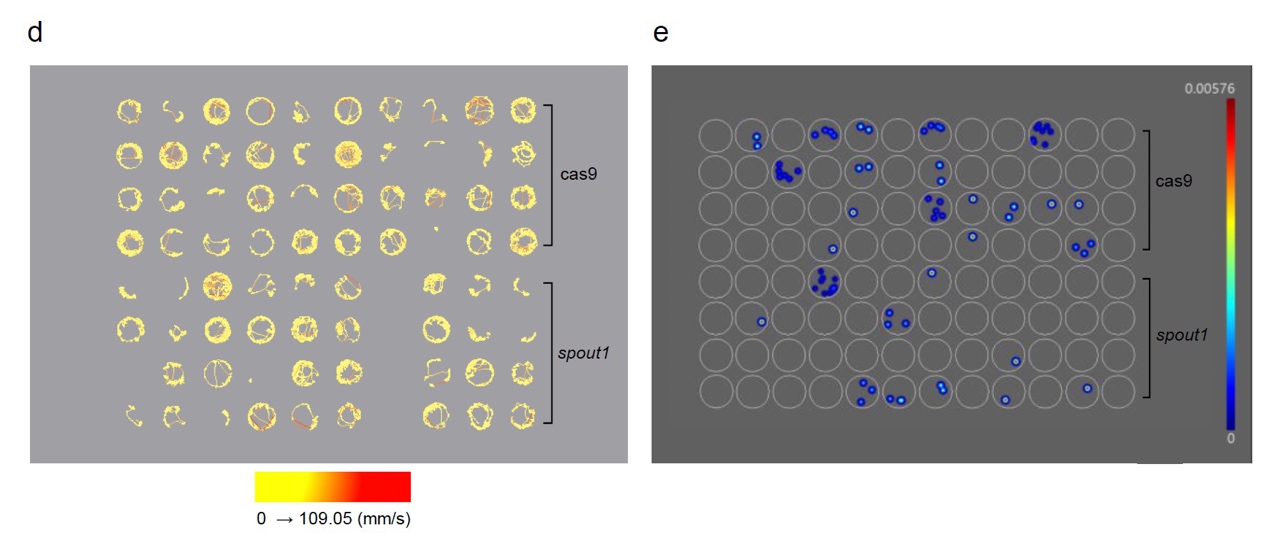


Fig. S10: Statistic analyses of spontaneous locomotor behavior between zebrafish in the cas9 control group and *spout1* knockout group in dark environment.


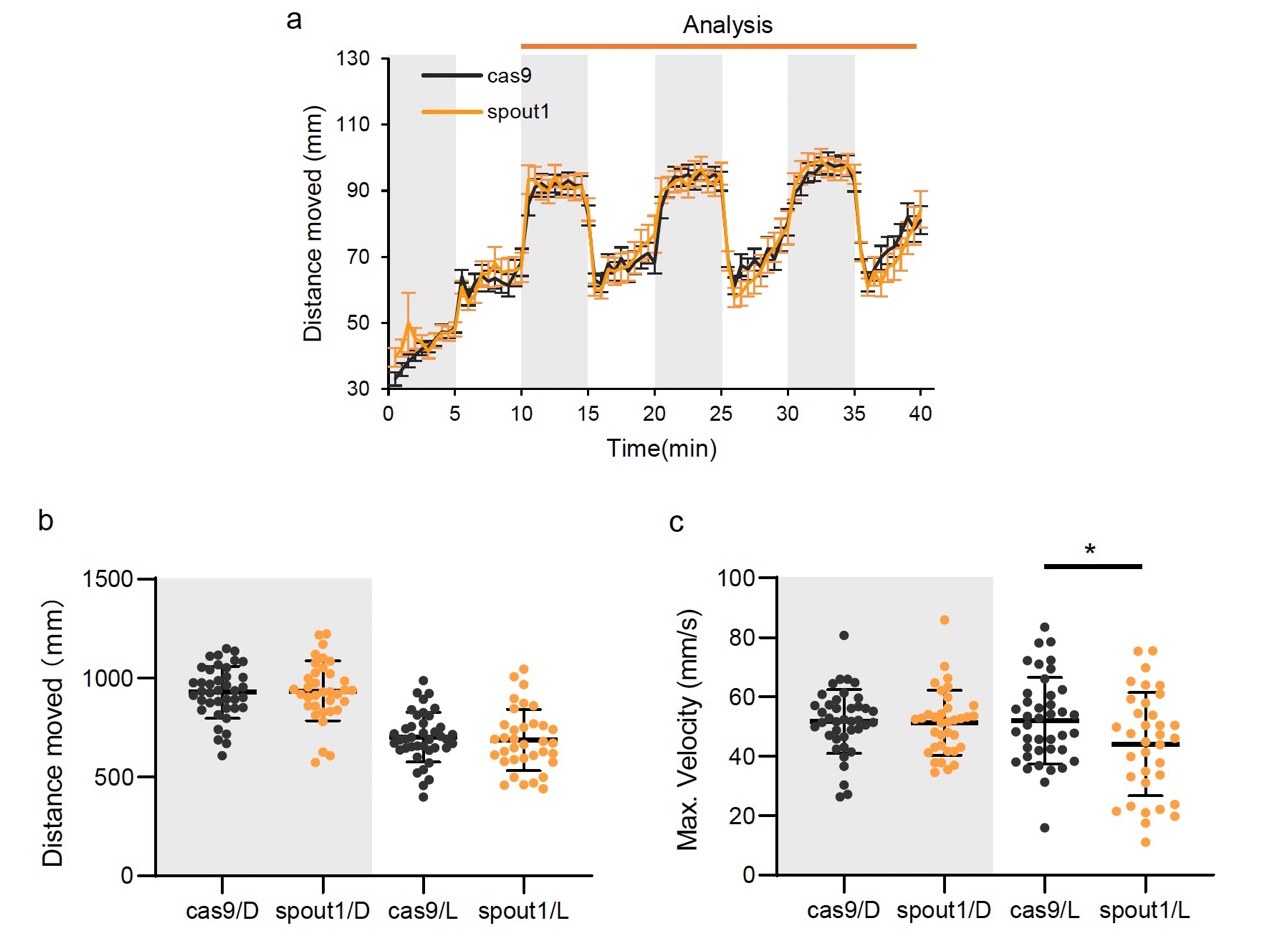


Fig. S11: Statistic analyses of distanced moved and max-swimming velocity between zebrafish in the cas9 control group and *spout1* knockout group in dark and light environment. (Unpaired *t* test, **p*-value=0.0348)

Fig. S12: Family tree and sanger sequencing data of patient 1.

Fig. S13: Family tree and sanger sequencing data of patient 2.

Fig. S14: Family tree and sanger sequencing data of patient 3.

Fig. S15: Family tree and sanger sequencing data of patient 4.
